# Ubiquitin signaling and the proteasome drive human DNA–protein crosslink repair

**DOI:** 10.1093/nar/gkad860

**Published:** 2023-10-16

**Authors:** Maram Essawy, Lisa Chesner, Duha Alshareef, Shaofei Ji, Natalia Tretyakova, Colin Campbell

**Affiliations:** Department of Pharmacology, University of Minnesota, Minnesota, MN 55455, USA; Department of Pharmacology, University of Minnesota, Minnesota, MN 55455, USA; Department of Pharmacology, University of Minnesota, Minnesota, MN 55455, USA; Department of Medicinal Chemistry, University of Minnesota, Minnesota, MN 55455, USA; Department of Medicinal Chemistry, University of Minnesota, Minnesota, MN 55455, USA; Department of Pharmacology, University of Minnesota, Minnesota, MN 55455, USA

## Abstract

DNA–protein crosslinks (DPCs) are large cytotoxic DNA lesions that form following exposure to chemotherapeutic drugs and environmental chemicals. Nucleotide excision repair (NER) and homologous recombination (HR) promote survival following exposure to DPC-inducing agents. However, it is not known how cells recognize DPC lesions, or what mechanisms selectively target DPC lesions to these respective repair pathways. To address these questions, we examined DPC recognition and repair by transfecting a synthetic DPC lesion comprised of the human oxoguanine glycosylase (OGG1) protein crosslinked to double-stranded M13MP18 into human cells. In wild-type cells, this lesion is efficiently repaired, whereas cells deficient in NER can only repair this lesion if an un-damaged homologous donor is co-transfected. Transfected DPC is subject to rapid K63 polyubiquitination. In NER proficient cells, the DPC is subject to K48 polyubiquitination, and is removed *via* a proteasome-dependent mechanism. In NER-deficient cells, the DNA–conjugated protein is not subject to K48 polyubiquitination. Instead, the K63 tag remains attached, and is only lost when a homologous donor molecule is present. Taken together, these results support a model in which selective addition of polyubiquitin chains to DNA-crosslinked protein leads to selective recruitment of the proteasome and the cellular NER and recombinational DNA repair machinery.

## Graphical Abstract

**Graphical Abstract:**
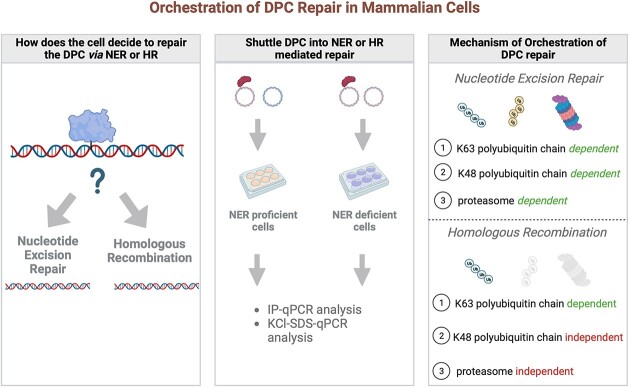

## Introduction

Enhanced DNA repair capability represents a potential avenue through which cancer cells become resistant towards DNA–protein crosslink (DPC) forming anti-neoplastic drugs, such as topoisomerase poisons, nitrogen mustard agents and platinum compounds (1–6). Despite recent interest in identifying the mechanisms of DPC repair, inherent heterogeneity of this type of DNA lesion represents a major challenge to these types of investigations (7). Because DPC adducts are far more structurally diverse than other types of DNA lesions (e.g. DNA double-strand breaks and UV photoproducts), bacteria, yeast, and higher eukaryotes have evolved multiple repair mechanisms that can be mobilized for the removal of different types of DPC lesions (8–15). Genetic and biochemical studies have established that some of the commonly formed DPCs, notably those containing topoisomerase and spo11, are subject to specialized hydrolysis-based and nucleolytic mechanisms, respectively. Other studies have shown that various DPCs formed upon exposure to formaldehyde, platinum-derived drugs, and topoisomerase poisons can be repaired by nucleotide excision repair (NER) and homologous recombination (HR) mechanisms (16–18). The regulatory mechanism(s) that orchestrate the cellular response to DPCs by these various pathways have not been fully elucidated. Several studies suggest that DPCs that are larger than 10–14 kDa in size are not amenable to NER, while HR machinery preferentially repairs DPCs comprised of larger proteins (8,9,11). Additionally, data generated in mammalian cells and in *Xenopus laevis* extracts suggest that HR can be tightly coupled to DNA replication, while NER is active at all phases of the cell cycle (19–25). Undoubtedly, there are types of DPCs that are subject to repair by either NER or HR, thus there is interest in understanding the events following recognition that determine the cellular fate of DPCs (i.e. repair by NER or HR).

Increasing evidence suggests that DPC repair is regulated by ubiquitin signaling. Sun *et al.* reported that co-treatment of mammalian cells with a ubiquitin ligase inhibitor and topoisomerase inhibitor resulted in an accumulation of topoisomerase-DPCs (26), suggesting that DPCs are ubiquitinated as part of the DPC repair response (26–28). Additionally, Liu *et al.* showed that 5-azadC treatment of cells that had been treated with siRNA targeting the Sumo Targeted Ubiquitin Ligase (StubL) RNF4 results in impaired ability of cells to repair DNA Methyltransferase I DPCs (27). Additional studies in *Xenopus* oocyte extracts have demonstrated that in a replication-dependent context, ubiquitination was associated with the recruitment of SPRTN metalloprotease and the proteasome to DPCs (25). Together, these findings are consistent with the interpretation that ubiquitin signaling modulates DPC repair *via* proteasome dependent and proteasome independent mechanisms.

A challenge in studying the mechanism of orchestration of DPC repair lies in the heterogeneity of xenobiotic-induced DPCs. Exposure to therapeutic drugs not only leads to the formation of numerous DPCs with varying sizes and structures but can also result in variation in the types of chemical bonds linking the DNA and protein molecules (4,29). This diversity poses a limitation because it becomes difficult to discern whether different DPCs are repaired through distinct mechanisms or if multiple modes of orchestration exist. To overcome this challenge, we query the role of ubiquitination in the repair of a site-specific DPC substrate formed on a non-replicating M13 molecule and introduced into mammalian cells. This model lesion enables us to specifically examine the mechanisms involved in the replication-independent repair of a homogenous and chemically defined DPC. Building upon our model system, we manipulated the DPC transfection conditions to selectively direct DPCs towards either NER or HR mediated repair pathways, allowing us to investigate the role of DPC ubiquitination in each pathway independently of the other. We employed an SSPE-qPCR assay developed in our laboratory (9) to monitor DPC repair, an antibody-based immunoprecipitation followed by qPCR to detect post translational modifications to the DPC, and a KCl-SDS based precipitation followed by qPCR to measure the removal of the crosslinked protein from DNA.

Based on the findings derived from these investigations, we constructed a working model that integrates the observed repair dynamics and ubiquitination patterns on DPC repair by NER and HR. Importantly, our model represents the first-ever demonstration of the mechanism of orchestration of repair of a DPC that is known to be subject to repair by NER and by HR. While previous studies have hinted at the involvement of ubiquitin in DPC repair, our findings propose a detailed mechanism for how ubiquitin signaling regulates the removal of DPCs. Notably, our model proposes that differential polyubiquitination leads to recruitment of the proteasome for the facilitation of repair of oversized DPCs by NER, or to recruitment of homologous recombination machinery without a requirement for proteasomal processing. This aspect of our study holds particular significance, as it adds a new dimension to the understanding of DPC repair and highlights the importance of ubiquitin in this process.

## Materials and methods

### Reagents

8-Oxo-2′-deoxyguanosine (8-oxo-dG) containing oligonucleotides were purchased from Midland Certified Reagent (Midland, TX). All other oligonucleotides were purchased from the University of Minnesota Genomic Center. Rabbit monoclonal anti-ubiquitin antibody was purchased from Abcam (Cat. #: ab134953, Lot #: GR3367020-2). 0.7 ug antibody was used in each ubiquitin pulldown. Rabbit polyclonal K48 linkage specific polyubiquitin antibody was purchased from Cell Signaling (Cat. #: 4289S, Lot #: 2). 1 ug antibody was used in each K48 linkage specific polyubiquitin pulldown. Mouse monoclonal K63 linkage specific polyubiquitin antibody was purchased from Biolegend (Cat. #: 932201, Lot #: B319879) 1 ul antibody was used in each K63 linkage specific polyubiquitin pulldown. Protein G beads were purchased from Invitrogen (Cat. #: 10003D), 5 ul beads were used for each IP. MG132 was purchased from Abcam (Cat. #: ab14707, Lot #: APN17146-2-1-S). Rabbit polyclonal anti-XPA primary antibody was purchased from Abcam (ab65963) and used at a 1:2000 dilution. HRP-conjugated goat-anti rabbit secondary antibody was purchased from Invitrogen (#31460) and used at a 1:10 000 dilution. Clarity Max ECL Western Blotting Substrate was purchased from Bio-Rad (#1705060).

### Biological resources

Human oxoguanine glycosylase 1 (OGG1) was expressed and purified from BL21(DE3) competent E. coli using a pET-28a expression vector. OGG1 R341 mutant was generated by restriction cloning of the OGG1 R341 cDNA sequence to replace the wildtype OGG1 cDNA sequence in this pET-28a expression vector. OGG1 K341R was then expressed and purified from BL21-Gold (DE3) competent cells. HEK293T cells were purchased from the American Type Culture Collection (ATCC) and cultured in Dulbecco's modified Eagle's medium (DMEM) supplemented with 9% fetal bovine serum (FBS). The XPD cell line is an immortalized dermal fibroblast cell line with a primary defect in the XPD gene, first isolated from a patient with Complementation Group D Xeroderma Pigmentosum. The XPD-C cell line is the gene-corrected derivative of the XPD cell line. Both cell lines (GM08207 and GM15877, respectively) were purchased from the Coriell institute and cultured in DMEM supplemented with 9% FBS. HT1080 WT and HT1080 XPA-KO cells were ordered from Synthego. Synthego used three gRNA sequences designed to target exon 1 of the human XPA gene. Analysis of the Sanger sequencing traces of HT1080 XPA-KO cells was performed using Synthego's open-source ICE too (synthego.com) and showed a 100% knockout score. HT1080 WT and XPA KO-KO cells were maintained in minimum essential medium eagle (MEM) supplemented with 9% FBS. MEF5 and MEF7 cells (isogenic murine cell lines wild-type and SPRTN-deficient, respectively, see below) were a generous gift from Yuichi J. Machida (30) and were maintained in Dulbecco's modified eagle medium (DMEM) supplemented with 9% fetal bovine serum (FBS).

### Construction of plasmid DNA repair substrates

Synthetic, 8-oxo-dG containing oligodeoxynucleotide (100 pmol) (M13-8oxo, or EcoRI-8oxo, Table 1) was phosphorylated with T4 PNK (40 units) in 1× DNA Ligase buffer for 30 min at 37°C. Phosphorylated oligonucleotide was then annealed to single-stranded M13 viral DNA (13.4 pmol) and extended with Taq polymerase (100 units), in a solution containing 1× Thermopol Reaction Buffer, 1× NEB Buffer 2, 100 mM ATP, 10 mM dNTPs, and 20 μg bovine serum albumin and incubated for 15 min at 75°C. The sample was cooled on ice, T4 DNA Polymerase (60 units) and T4 DNA Ligase (8000 units) were added, and the sample was incubated at 37°C overnight for complete extension and ligation of the new double stranded M13 DNA containing an 8-oxo-dG residue. Following overnight incubation, 8-oxo-dG containing DNA was purified by phenol-chloroform extraction and ethanol precipitation. To make plasmid DPC (Supplemental Figure 1), 8-oxodG containing M13 DNA was crosslinked to OGG1 in 200× or 250× protein: DNA molar excess (for crosslinking of recombinant wild type OGG1, or recombinant OGG1 K341R, respectively) in buffer containing 100 mM NaCl, 1 mM MgCl_2_, 20 mM Tris–HCl pH 7.0, and 10 mM sodium cyanoborohydride. Sodium cyanoborohydride stock solution was made fresh, and added immediately before OGG1, which was added last to the reaction mixture. Crosslinking was carried out at 37°C for 1 h. During the crosslinking reaction, lysine residue 249 of OGG1 nucleophilically attacks C1, the attachment site of the deoxyribose sugar in the DNA backbone to 8-oxo-dG. This attack causes removal of 8-oxo-dG and the creation of an apurinic site in the DNA. The presence of sodium cyanoborohydride in the reaction causes the reduction of the Schiff base intermediate formed by OGG1 during this reaction, covalently trapping OGG1 to C1 of the deoxyribose.

**Table 1.** Oligodeoxynucleotides (ODNs) used in this study. 8-oxoguanine (8-oxo0dG) containing sequences were annealed to M13mp18 and then extended using Taq polymerase to make double stranded, 8-oxoguanine containing M13, which was then crosslinked to OGG1 to make the DPC substrate

ODN	Sequence	Use
**M13-8oxo**	5′-AGGGTTTTCCCA(8-oxo-dG)TCACGACGTT-3′	Primer Extension
**EcoRI-8oxo**	5′-CCGGGTACCGAGCTC(8-oxo-dG)AATTCGTAATCTTGGTCATAGCTG-3′	Primer Extension
**Fragment b L**	5′-CACCCCAGGCTTTACACTT-3′	qPCR of M13 Plasmid
**Fragment b R**	5′-GTAAAACGACGGCCAGTG-3′	qPCR of M13 Plasmid
**Fragment c L**	5′-CTGGGTGCAAAATAGCAACT-3′	qPCR of M13 Plasmid
**Fragment c R**	5′-CCCAATAGCAAGCAAATCA-3′	qPCR of M13 Plasmid
**DPC SSPE L**	5′-GCTGCAAGGCGATTAAGT-3′	SSPEqPCR of M13 Plasmid
**DPC SSPE R**	5′-CGGCTCGTATGTTGTGTG-3′	SSPEqPCR of M13 Plasmid

See text for details. Depicted oligonucleotides were used for qPCR analyses. Fragment b and c respectively represent the amplicons found in the red and black M13 fragments depicted in the schematics shown in Figure 2A and Figure 3A. DPC SSPE represents the amplicon surrounding the DPC, depicted in the schematic shown in Supplemental Figure 1.

### Transfection of DPC into cells

Sixteen hours before transfection, cells were counted and plated into six-well plates at a density of 600 000 cells per well. In the case of drug pretreatment, culture media was removed from the well by gentle aspiration and replaced with 4 mL serum-free media (SFM) ± drug. MG132 was used at 10 uM concentration for 1 h prior to transfection. BO2 was used at 5 uM concentration for 1 h prior to transfection. Following drug pretreatment, drug containing media was removed by gentle aspiration and replaced with DMEM containing 9% FBS. For DPC transfection, 1 ug of plasmid DPC was added to each well in combination with lipofectamine, following manufacturer instructions. Plates were incubated at 37°C. 30 min following the beginning of transfection, media and lipofection mixture were removed from the well by gentle aspiration and replaced with 4 ml DMEM supplemented with 9% FBS, and plates resumed incubation at 37C until cell collection at predetermined time points. At these time points, cells were lysed, and transfected DNA was collected by a modified HIRT procedure. Briefly, in each well, media was removed by gentle aspiration and replaced with 1 ml of lysis buffer containing 0.6% SDS and 0.01 M EDTA. Plates were incubated for 10 min at room temperature, then cells were scraped by rubber policeman and transferred into a 1.5 ml Eppendorf tube. NaCl was added to a final concentration of 1M, tubes were closed and inverted sharply 10 times, and incubated at 4°C overnight. The next day, tubes were spun in a tabletop centrifuge at 21 000 × g at 4C for 30 min. Pellets were discarded and DNA was collected from supernatant by ethanol precipitation.

### Immunoprecipitation and PCR analysis

DNA recovered from transfected cells by HIRT procedure was digested with 40 units HhaI at 37C for 1 h. Following HhaI restriction digest, samples were mixed with 200 ul IP Buffer (25 mM Tris–HCl pH 8.0, 150 mM NaCl, 1 mM EDTA, 1% NP-40, 2.5% (w/w) BSA 250 pmol of recombinant OGG1). 40 ul of this mixture was removed and saved as ‘input control’. To the remaining 200 ul, antibody for pulldown was added, and samples were placed on a tube inverter at 4°C for 3 h. For each antibody pulldown, 5 ul of protein G Magnetic beads were incubated with 200 ul Blocking Buffer (25 mM Tris–HCl, pH 8.0, 150 mM NaCl, 1 mM EDTA, 1% NP-40, 5% (w/w) BSA, 250 pmol recombinant OGG1), and inverted at 4C for 3 h. Following 3-h intubation, protein G solution was combined and incubated with DNA/antibody/IP buffer containing sample at 4°C for 1 h. To collect protein G bound material, tubes were placed on a magnetic separation rack and protein G beads were washed 3× with Washing Buffer 1 (25 mM Tris–HCl, pH 8.0, 150 mM NaCl, 1 mM EDTA, 1% NP-40 and 2.5% (w/w) BSA), 2× with Washing Buffer 2 (25 mM Tris–HCl, pH 8.0, 150 mM NaCl, 1 mM EDTA), and 2× with 1× TE buffer. DNA was recovered from beads by incubation with Proteinase K (8 units) in Proteinase K Buffer (10 mM Tris–HCl, pH 8.0, 1 mM EDTA, 0.5% SDS), for 1 h at 37C, and finally by QIAquick PCR purification kit (following manufacturer protocol). 1 ul of PCR purified DNA was then added to 27 ul DI H2O, 30 ul 2X SYBR Green Master Mix, and 100 pmol of each primer. (DNA was amplified by two different primer sets in parallel: for detection of DPC containing fragment, primer pair Fragment b L and Fragment b R (Table 1) were used, and for detection of control fragment, primer pair Fragment c L and Fragment c R (Table 1) were used. All samples were loaded in duplicate onto a 96 well plate and Real time PCR was performed using the Applied Biosystems StepOnePlus Real Time PCR System under the following conditions: 10 min denaturing at 95C (1×), then 30 s denaturing at 95°C, 30 s primer annealing at 57°C, and 30 s for Taq polymerase extension at 72°C (40×).

### KCl-SDS precipitation

Following HhaI restriction digest, SDS was added at a concentration of 0.5% to DNA recovered from transfected cells, then the samples were heated at 65C for 10 min. KCl was then added to 100 mM final concentration, and tubes were chilled on ice for 5 min. Next, tubes were centrifuged at 21 000 × g, for 5 min at 4°C. Supernatants were carefully transferred to another tube using a gel loading pipette tip, and centrifugation was repeated. Supernatants were again transferred to a new tube using a gel loading pipette tip, diluted, and analyzed by qPCR, under the same primer and amplification conditions used for qPCR analysis of IP samples.

### SSPE-qPCR

DNA recovered from transfected cells by HIRT prep then subjected to SSPE-qPCR as described previously. Briefly, DNA was added to 22.5 ul DI H2O, 30 ul 2× SYBR Green Master Mix, and 100 pmol DPC F primer (Table 1) into each of two tubes. One tube was placed on ice while the other tube was subjected to eight rounds of strand specific PCR (utilizing 100 pmol of DPC F primer). Next, 100 pmol DPC R primer (Table 1) was added to the tube that underwent the eight rounds of strand specific PCR, and to the tube that was incubated on ice. All samples were then loaded in duplicate onto a 96-well plate and Real time PCR was performed using the Applied Biosystems StepOnePlus Real Time PCR System under the following conditions: 10 min denaturing at 95C (1×), then 30 s denaturing at 95°C, 30 s primer annealing and Taq polymerase extension at 60°C (30×). Cycle Threshold (Ct) values were determined by Applied Biosystem StepOnePlus Software. Delta Ct values were determined by subtracting the Ct of the sample that underwent 8 cycles of Strand Specific PCR from the Ct of the same sample that, in parallel, did not undergo 8 cycles of Strand Specific PCR. Percent undamaged DNA was calculated by using the formula (percent undamaged = 2^Delta Ct^/2^3^ × 100) and percent repair was calculated using the formula [percent repair = (percent undamaged Tx) – (percent undamaged to)].

### Western blot

Cells were lysed using 1× RIPA lysis buffer supplemented with protease inhibitors. Cell lysate was rotated for 30 min at 4C, then spun at 12 000 rpm for 20 min. Bicinchoninic acid (BCA) assay was used to determine protein concentration, and 20 ug of protein was loaded on a Bolt Bis–Tris Plus 12% gel for western blotting using rabbit anti-XPA primary antibody and an HRP-conjugated goat anti-rabbit secondary antibody. HRP chemiluminescence was detected using Clarity Max ECL Western Blotting Substrate, following manufacturer's protocol and imaged using a Bio-Rad ChemiDoc Imaging System.

### Statistical analyses

**Figure 1.**
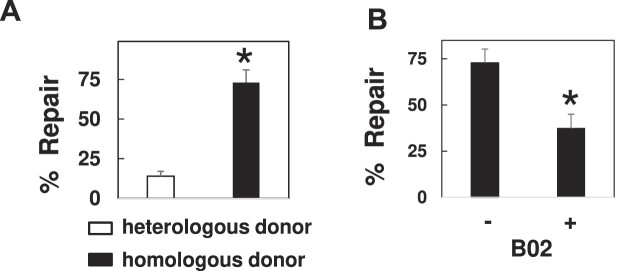
DPCs are subject to repair by HR. (**A**) DPCs were transfected into NER deficient cells (XPD), in the presence of a heterologous (white bar) or homologous (black bar) donor molecule. Low molecular weight DNA was recovered 3 h post-transfection and subjected to the SSPE-qPCR repair assay. *P= 0.01. (**B**) DPCs were transfected into untreated (–) or B02 pre-treated (+) XPD cells. Low molecular weight DNA was recovered 3 h following transfection and subjected to the SSPE-qPCR repair assay. *P= 0.04.

**Figure 2.**
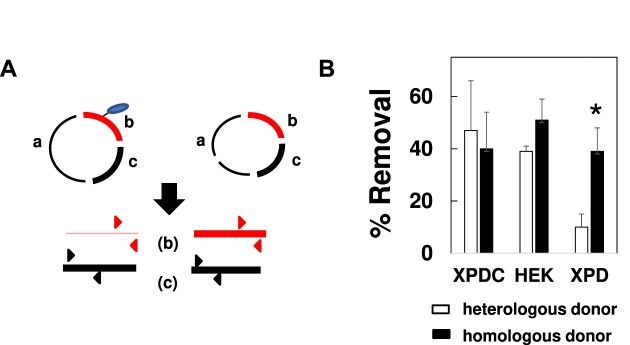
Removal of DNA-linked protein during DPC repair *via* NER and HR. (**A**) Schematic of KCl-SDS-qPCR assay. KCl and SDS are used to selectively precipitate protein-crosslinked DNA. qPCR is then used to quantify the abundance of the DPC-containing fragment (red) relative to the control, non DPC-containing fragment (black). See text for details. (**B**) DPCs were transfected into cells proficient for NER (XPDC, HEK) or deficient for NER (XPD), in the presence of a heterologous or homologous donor plasmid. DPCs were recovered 1-h post-transfection and subjected to KCl-SDS-qPCR. Results depict % removal, measured by qPCR quantification of DPC-containing DNA, prior to and following transfection. *P= 0.01.

**Figure 3.**
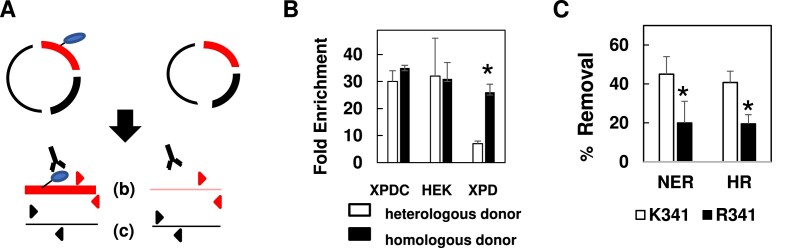
DPCs are ubiquitinated in NER and HR-mediated repair. (**A**) Schematic of anti-ubiquitin IP-qPCR. Anti-ubiquitin antibody is used to specifically enrich for ubiquitin (and any covalently bound protein or DNA). qPCR is then used to quantify the abundance of the DPC-containing fragment (red) relative to the control, non DPC-containing fragment (black). See text for details. (**B**) DPCs were transfected into cells proficient for NER (XPDC, HEK) or deficient for NER (XPD) in the presence of a heterologous or homologous donor plasmid. Low molecular weight DNA was recovered from cells one-h post transfection and subjected to anti-ubiquitin IP-qPCR. Results depict fold-enrichment of DPC containing fragment following antibody treatment, measured by qPCR, *P= 0.02. (**C**) DPC substrates produced by cross-linking the wild-type OGG1 protein (K341) or arginine for lysine-substituted version (R341). DPCs were transfected into HEK293T cells in the absence of homologous donor into NER proficient cells (NER) or in the presence of homologous donor into NER deficient cells (HR) cells. Low molecular weight DNA was recovered 1 h following transfection. DPCs were then subjected to KCl-SDS-qPCR, as described above, to determine the percentage of crosslinked protein removed (% Removal), *P= 0.05.

**Figure 4.**
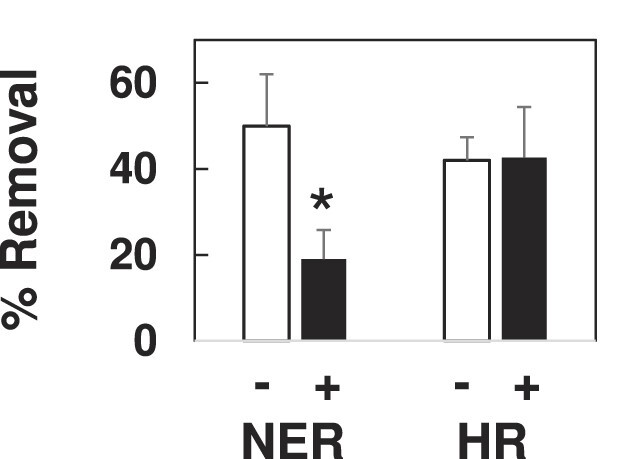
Effect of proteasome inhibition on DPC removal mediated *via* NER and HR. NER-mediated removal was measured by transfecting DPC substrate into XPDC cells in the absence of a homologous donor, and HR-mediated removal was measured by transfecting DPC substrate into XPD cells in the presence of a homologous donor. Transfections were performed on cells that had been pre-treated for 1-h in the absence (–) or presence (+) of 10 μM MG132. Low molecular weight DNA was recovered one-h post-transfection, subjected to KCl-SDS-qPCR and % removal of DPC determined as described in the legend to Figure 2. *P= 0.05.

**Figure 5.**
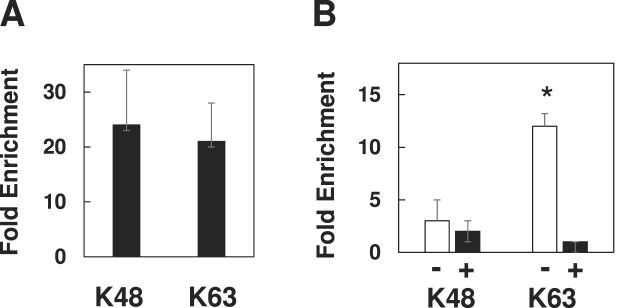
Differential polyubiquitination patterns during NER and HR mediated repair. (**A**) DPCs were transfected into NER-proficient HEK293T cells and low molecular weight DNA recovered one-h post-transfection, then subjected to IP-qPCR using anti-K48 or K63 polyubiquitin -selective antibodies. (**B**) DPCs were transfected into NER-deficient XPD cells along with a non-homologous (–) or homologous (+) donor molecule and IP-qPCR performed as described above.

Data in Figures 1–5 are represented as mean values, and error bars represent standard error of the mean. Experiments done to generate the data represented in Figure 1A and B were repeated 3 and 5 times, respectively. Experiments done to generate the data represented in Figures 2–5, as well as Supplemental Figures 3–4, were each performed in triplicate. In each ‘repeat’ noted above, 1 ug of DPC substrate was freshly generated *in vitro*, transfected into one well of seeded mammalian cells, recovered and subjected to qPCR-based analysis one time. *P*-values for Figures 1A–B, 2B, 3B and 5A–B were calculated using a one-tailed *t*-test, while *P*-values for Figures 3C and 4 were calculated using a two-tailed homoscedastic *t*-test. One-tailed *t*-tests were done when transfected samples were compared to untransfected samples while two-tailed *t*-tests were done when transfected samples were compared to other transfected samples.

## Results

### DPCs are subject to repair by HR

We and others (9,11) have previously shown that substrates containing DPC lesions are efficiently repaired following introduction into mammalian cells, and that the efficiency of repair is substantially lower in cells deficient in NER activity. Reports have shown that bacterial, yeast and mammalian cells harboring defects in HR genes display an impaired tolerance for DPCs (8,13,31), suggesting that the DPC repair defect observed in NER-deficient cells could be reversed if an undamaged homologous donor molecule were co-transfected with the DPC-containing substrate. To test this hypothesis, we transfected a DPC substrate containing a single human oxoguanine glycosylase I (OGG1) protein molecule cross-linked to double-stranded M13 (see (9)) into an immortalized NER-deficient cell line (GM08207 [38]) derived from a human donor harboring inactivating mutations in the XPD gene. The DPC was transfected in the presence of either un-lesioned M13 (a homologous donor) or a plasmid with no sequence similarity to M13 (a heterologous donor). Low molecular weight DNA was recovered from the NER-deficient cells (hereafter referred to as XPD) three-h post-transfection, and the percentage of repaired substrates present determined using a strand-specific primer extension/quantitative real-time PCR assay (termed SSPE-qPCR, as outlined in Supplemental Figure 2 and described in reference (9)). We observed that 73% of DPCs were repaired in XPD cells co-transfected with the homologous donor, whereas only 14% repair was observed when these cells were co-transfected with the heterologous donor molecule (Figure 1A). These results convincingly argue that the OGG1-containing DPC lesions are subject to repair *via* HR.

During HR repair, the 5′ ends of DNA are subject to endonucleolytic digestion to generate 3′ single-stranded DNA ends (32,33). This single-stranded DNA is initially coated by DNA replication protein A (RPA) and activates the ATR (Ataxia Telangiectasia and Rad3-related protein) response (34). A Rad51 nucleofilament is then assembled and replaces the RPA coat to initiate the search for a homologous donor (35–37). We therefore reasoned the NER-independent repair depicted in Figure 1A would be lost in cells that have impaired Rad51 activity. To test this prediction, XPD cells were pre-treated for one h in the presence or absence of 5 μM B02 (38), a pharmacological inhibitor of Rad51. Following B02 pretreatment, DPCs were transfected into cells in the presence of homologous donor and collected 3 h following transfection, then DPC repair assayed as outlined above. The results presented in Figure 1B indicate that pretreatment with B02 resulted in a two-fold reduction in DPC repair in XPD cells. Together, the results presented in Figure 1, combined with our previous results [20] indicate that human cells can efficiently repair a DPC lesion *via* either the NER or HR pathways.

### DPC removal in NER deficient cells, but not in NER proficient cells, is dependent on homologous donor

This finding raises the fundamental question of how cells determine which repair pathway, e.g. HR or NER, is recruited to repair a DPC lesion. In cells undergoing replication and transcription, the context in which a particular DPC lesion is detected may dictate the outcome. For example, DPC lesions encountered at replication forks are likely to be subjected to HR-mediated repair (20,21), whereas lesions detected during transcription are likely to be targeted for transcription-coupled NER (9,39). However, the repair substrate utilized in these experiments is subject to neither replication nor transcription. The observation that this synthetic DPC substrate can be repaired *via* either mechanism led us to ask whether examining early repair intermediates could provide insight into the decision-making process.

The SSPE-qPCR repair assay described above is unsuitable to address this question, because it only detects the fully repaired product. We therefore employed a KCl/SDS technique (40) which selectively precipitates DPC containing DNA, to examine removal as the DPC substrate undergoes either recombination or NER-mediated repair. In this way, we were able to quantitatively assess the extent to which the cross-linked OGG1 protein has been removed from the M13 molecule (See Figure 2A). Briefly, low molecular weight DNA recovered from transfected cells was digested with *HhaI* restriction endonuclease and subsequently subjected to KCl/SDS precipitation. The supernatant material was then subjected to parallel qPCR analyses using, in one instance, a primer pair specific for a restriction fragment (denoted ‘b’ in Figure 2A) that includes the protein crosslink site, and, in the other, a second primer pair specific for a restriction fragment (denoted as ‘c’ in Figure 2A) from a different, undamaged region of M13. The cycle thresholds (Ct) of these respective qPCRs can be used to calculate the relative abundance of these two restriction fragments, which can, in turn, be used to calculate the percentage of recovered molecules from which the DPC has been removed, which we depict as ‘% Removal’ (see MAterials and Methods section for details).

Based on the results presented in Figure 1, we predicted that DPC removal would occur in the absence of homologous donor in NER proficient cells, whereas removal of the crosslinked protein in NER-deficient clones would require the presence of a homologous donor. To test this hypothesis, DPCs were transfected into XPD cells in the presence or absence of homologous donor, as well as into a gene-corrected derivative of this line (GM15877, referred to hereafter as XPDC), and a second NER-proficient cell line, HEK293T. Low molecular weight DNA was recovered one-h post-transfection, and DPC removal was quantified by KCl/SDS-qPCR as described above. The results presented in Figure 2B show that DPCs transfected into NER proficient cells were removed efficiently in both the presence and absence of a homologous donor. In contrast, DPC removal in the XPD cells co-transfected with the heterologous donor were significantly lower than those observed in XPDC and HEK cells. In contrast, co-transfection of homologous donor with the DPC substrate led to protein removal at levels indistinguishable from those observed in the NER-proficient cells. To confirm that the observation made in Figure 2B is not specific to the XPD complementation group defect, experiments were repeated into wild-type HT1080 cells as well as in a line derived from them in which CRISPR/cas9 targeting was used to introduce inactivating mutation in the XPA gene. Results from these experiments revealed that, unlike their wild-type counterparts, cells lacking a functional XPA gene were unable to efficiently remove DPC in the absence of an undamaged homologous donor-molecule (Supplemental Figure 3).

### Ubiquitination is required for efficient DPC removal in both NER and HR pathways

It has been shown that xenobiotic-induced chromosomal DPCs are subject to ubiquitin conjugation shortly following their formation (26–28). To examine the role of ubiquitination in removal of M13-crosslinked OGG1 protein we developed the IP-qPCR assay outlined in Figure 3A. This assay resembles the KCl/SDS-qPCR assay in that the assay is initiated by restriction endonuclease digestion of low molecular weight DNA recovered from cells transfected with the DPC substrate.

However, rather than using KCl/SDS-based precipitation to deplete DPC containing fragments, an anti-ubiquitin antibody is used to selectively *enrich* for ubiquitinated DPC-containing fragments. Immuno-affinity purified material is recovered from protein G beads by digestion with proteinase K and qPCR amplification is performed using the same primer pairs depicted in Figure 1 (see in the Materials and Methods section for details). The antibody-based capture results in selective enrichment of ubiquitin-conjugated material, and subsequent qPCR analysis allows for the quantitative detection of recovered DNA. In these experiments, data are expressed as ‘Fold Enrichment’, which indicates the relative abundance of the DPC-containing fragment compared to the control fragment. We used this assay to measure DPC ubiquitination levels under conditions when the substrate is subject to primarily NER-mediated repair (when DPC substrate is transfected into HEK293T or XPDC cells in the absence of homologous donor) or HR-mediated repair (when substrate is transfected into XPD cells in the presence of homologous donor DNA). The results presented in Figure 3B indicate that DNA-crosslinked OGG1 becomes ubiquitinated following transfection into NER-proficient cells in both the presence and absence of homologous donor. In contrast, in the NER-deficient XPD cells, substantial levels of OGG1 ubiquitination were only observed when an undamaged homologous donor molecule was present. The observation that DPC ubiquitination was only detected under cellular conditions that facilitated DPC repair was striking, and consistent with the interpretation that DPC ubiquitination is an intermediate step in DPC removal/repair.

To test the hypothesis that DPC ubiquitination is required for repair, we expressed and purified a recombinant OGG1 protein that contains a lysine to arginine substitution at lysine 341 (K341). This residue was chosen for modification because it has been shown that substitution of this residue suppressed OGG1 ubiquitination *in vitro* (41). Utilizing this strategy (i.e. blocking DPC ubiquitination by modification of a known ubiquitination site on the crosslinked protein) rather than using a pharmacological inhibitor of ubiquitination would allow us to directly query the role of ubiquitination of the DNA-crosslinked protein, and not of global cellular ubiquitination, in DPC repair. To confirm that DPCs generated with the OGG1 containing the R341 modification were less efficiently ubiquitinated, DPC substrates comprised of either unmodified (K341) OGG1 or modified (R341) OGG1 were transfected into HEK293T cells in the absence of homologous donor or into XPD cells in the presence of homologous donor, and low molecular weight DNA recovered one h later and subjected to anti-ubiquitin IP-qPCR. In both cases, we found that DPCs generated using R341 OGG1 were subject to ubiquitination at levels two-fold lower than DPCs generated using K341 OGG1 (data not shown).

DPC substrates comprised of either unmodified (K341) OGG1 or modified (R341) OGG1 were transfected into HEK293T cells in the absence of homologous donor (to monitor NER-mediated DPC repair), or into XPD cells in the presence of homologous donor (to monitor HR-mediated DPC repair), and low molecular weight DNA recovered one h later. This material was then subjected to KCl-SDS-qPCR to examine the effect of DPC ubiquitination on DPC removal. As depicted in Figure 3C, during NER-mediated repair, the R341 version of OGG1 was significantly less well removed (20% removal) than was the native, K341 OGG1 protein (45% removal). Similarly, during HR-mediated repair the R341 protein variant was less efficiently removed than K341 (20% and 40% removal, respectively) one-h post-transfection into XPD cells in the presence of homologous donor, indicating that DPC removal by both NER and HR is at least partially dependent on ubiquitination of the DNA-crosslinked protein.

### DPC removal by NER, but not HR, is proteasome dependent.

The results presented in Figure 3 suggest that ubiquitination may trigger proteasomal degradation of DNA-crosslinked OGG1 as part of the repair mechanism. The available evidence suggests that the NER machinery is only capable of repairing DPC comprised of relatively small peptides or proteins, however no such limitation has been associated with HR-mediated DPC repair (see Introduction). We therefore predicted that pre-treatment with the proteasome inhibitor MG132 would differentially impact DPC removal, dependent on which repair pathway was responsible for the repair. To test this hypothesis, cells were pre-treated for one h in the presence or absence of MG132 prior to transfection with DPC containing plasmid. As depicted in Figure 4, DPC removal mediated *via* NER (DPC transfected into XPDC cells with heterologous donor) was decreased two-fold in MG132-treated cells relative to non-treated cells. In contrast, proteasome inhibition had no effect on the efficiency of DPC removal during HR-mediated repair (DPC transfected into XPD cells with homologous donor). To test the hypothesis that DPC repair is facilitated by SPRTN protease, we transfected DPCs into embryonic fibroblast cells from wildtype mice (MEF5) or from SPRTN gene-mutated mice that harbored one null SPRTN allele and one ‘floxed’ allele (MEF7) (30). KCl-SDS-qPCR analysis of DPCs recovered 1 h following transfection showed that SPRTN deficiency had no effect on DPC removal (Supplemental Figure 4), suggesting that SPRTN does not play a role in replication-independent DPC repair.

### DPCs are differentially polyubiquitinated in NER vs HR repair backgrounds

In addition to triggering proteasomal degradation, ubiquitination can serve as a signal for a number of additional fates. While post-translational modification of proteins with a single ubiquitin protein (monoubiquitin) is known to serve several signaling purposes in cells including protein sorting and trafficking (42–44), ubiquitin can also be conjugated through one of its seven lysine residues to form polyubiquitin chains (45). Interestingly, it has been previously shown that topoisomerase-1 and topoisomerase-2 DPCs formed following treatment of mammalian cells with camptothecin or etoposide are modified with these chains (26). K48-linked polyubiquitin chains trigger proteasomal degradation of cellular proteins (46), while K63-linked polyubiquitin chains act as a signal for a variety of processes, including the recruitment of DNA damage response proteins (47,48). Therefore, we predicted that during NER repair, DPCs would undergo K48-linked polyubiquitination, and that in contrast, during recombinational repair DPCs would undergo K63-linked polyubiquitination. To test this hypothesis, DPCs were transfected into NER-deficient or proficient cells and low molecular weight DNA recovered one-h post-transfection. Following recovery, DPCs were subjected to anti-K48 or anti-K63 polyubiquitin-specific IP-qPCR. As Figure 5A illustrates, during NER-mediated repair the DNA-crosslinked OGG1 protein is subject to both K48 and K63 polyubiquitination. In contrast, when the DPC substrate was introduced into XPD cells in the presence of a homologous donor molecule neither K48 nor K63 polyubiquitination was detected (Figure 5B). Interestingly, replacement of the homologous donor with a heterologous molecule resulted in robust levels of K63 polyubiquitination (Figure 5B). Notably, in this latter instance, we still failed to detect K48 polyubiquitination of the DNA-crosslinked OGG1 protein.

Together, these findings are consistent with the interpretation that K48-linked polyubiquitination is triggered by the presence of NER machinery at the DPC, and that K63-linked polyubiquitination modulates DPC removal by HR.

## Discussion

**Figure 6.**
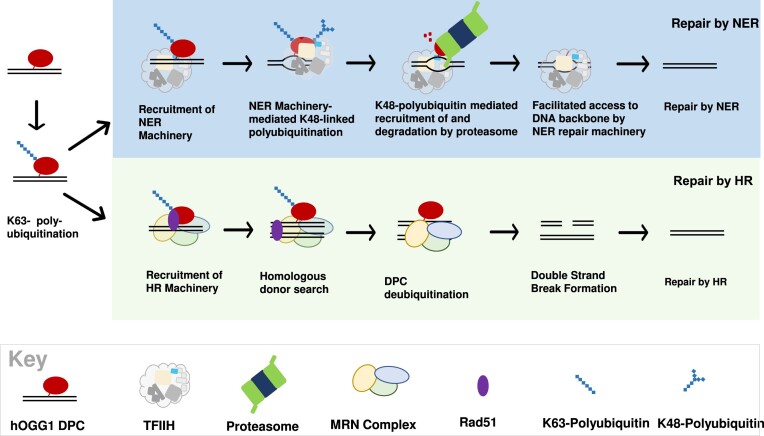
Proposed model of DPC ubiquitination and repair. Following recognition of a DPC lesion by NER machinery, DPCs are modified with K48- linked polyubiquitin chains. K48-polyubiquitination of DPC triggers proteasomal recruitment to and degradation of the crosslinked protein, eliminating steric hindrance by the DPC and allowing access to the DNA backbone by NER machinery. In the absence of NER machinery, DPCs are K63-polyubiquitinated. HR machinery are recruited to K63-polyubiquitinated DPCs and initiate a homologous donor search resulting in nucleolytic DPC repair by HR.

Cells utilize both NER and HR to repair DPCs, however considerable uncertainty exists regarding how one or the other pathway is selected to target individual lesions. To address this issue, we developed a simplified system in which a model DPC molecule is transfected into human cells, recovered and subjected to analysis. We show that while the substrate can be efficiently repaired by either pathway, manipulation of the system can dramatically influence the mechanism though which DPC repair occurs. In this way, we identified repair pathway-selective covalent modifications to the DNA-crosslinked protein. Our results lead us to propose a working model (depicted in Figure 6) of cellular orchestration of DPC repair. The key features of this model are discussed below. It is important to note that this model of DPC repair is likely to be relevant to lesions in loci that are not currently undergoing active replication or transcription. We hypothesize that specialized sub-pathways of DPC repair are likely to be involved in the processing of lesions detected under these circumstances.

### Ubiquitination as an essential DPC recognition event

In this model, DPC recognition triggers ubiquitination of the DNA-crosslinked protein, and this post-translational modification targets the cellular DNA repair machinery to the lesion. Consistent with this prediction, our data show that ubiquitination is an essential step, since converting a key residue (lysine 341) to arginine dramatically reduces the efficiency of both NER and recombinational repair. This finding builds on previous reports showing that pharmacological inhibition of cellular ubiquitination impairs xenobiotic-induced DPC repair (49–53), and demonstrates that the DNA-crosslinked protein must be ubiquitin-modified to ensure efficient removal. The finding that K63 polyubiquitination is associated with both NER and recombinational DPC repair leads us to propose that K63 polyubiquitination of the crosslinked protein is associated with recruitment of both NER and HR machinery. It is noteworthy that while the model predicts that this K63 polyubiquitination represents the initial DPC recognition event, additional experimentation will be required to confirm this, and it remains quite possible that DPCs are subject to additional, yet to be discovered, forms of modification.

### Differential polyubiquitination drives DPC repair fate

In contrast to K63 polyubiquitination signal, our results indicate that K48 polyubiquitination is only seen during NER-mediated DPC repair. We consequently incorporate into our working model the prediction that this modification commits the lesion to repair *via* NER. While the precise molecular mechanism remains obscure, it is known that p44, one of the 5 non-helicase subunits of TFIIH, is a homolog of the yeast Ssl1, a molecule that is known to possess E3 ligase activity (54). We therefore propose that TFIIH-mediated K48 polyubiquitination triggers proteasome-mediated processing of the DPC lesion, leading ultimately to the creation of a DNA-peptide substrate small enough to be subject to NER. Consistent with this hypothesis Baker *et al.* (11) have shown that the NER machinery is able to excise small DNA-crosslinked peptides, but not full-sized DPCs. Further supporting this prediction is the finding that the multiprotein TFIIH complex facilitates the opening of DNA around an NER substrate via an XPD and XPB-dependent mechanism (55,56).

Our results show that M13-crosslinked OGG1 transfected into NER-deficient cells in the absence of an undamaged homologous donor is not repaired. Instead, the DPC recognition/processing steps stall at the stage of initial K63 linked polyubiquitination step. However, when an undamaged homologous donor is co-transfected, the DNA-crosslinked protein is removed, and the lesion is subsequently repaired in a proteasome-independent process. We therefore propose that during recombinational repair, nucleolytic processing at the DPC site results in the creation of a DNA double strand break (14,57), however, this prediction remains to be experimentally verified.

In this model, we propose that NER is the ‘default’ repair pathway and that recombinational repair is only employed when this option is not available. As discussed above, NER activation is associated with the K48 polyubiquitin modification of the DNA crosslinked protein. In contrast, DPC substrates subject to recombinational repair are not modified in this way. At this time the molecular mechanism(s) through which the HR machinery removes the DPC are unclear. Our data show, however, that removal of the DPC requires the presence of a suitable homologous donor molecule. This hierarchical model is consistent with our findings that co-transfection of undamaged homologous donor DNA does not further enhance DPC removal in NER-proficient cells. An attractive aspect of the model is that it provides a plausible strategy through which cells can avoid generating potentially mutagenic and cytotoxic DNA double strand breaks by prioritizing NER over HR in repairing DPCs outside the context of replication. Again, as was mentioned above, additional experimentation will be required to confirm, and further refine this model.

### DPCs can undergo proteasome-dependent or independent processing

In NER-mediated repair, the addition of the K48 polyubiquitin chain to the crosslinked protein is postulated to recruit the proteasome, leading to proteolytic processing, converting the DPC to a DNA-crosslinked peptide that is subsequently repaired by the cellular NER machinery. This feature of the model is consistent with reports that repair of xenobiotic-induced chromosomal DPCs and DPCs generated *in vitro* is proteasome-dependent, and that only DPCs smaller than ∼10–14 kDa are amenable to repair by NER (11,58). The relatively large size of the DNA-crosslinked OGG1 (∼37 kDa) is proposed to sterically hinder initiation of NER by incision of the DNA around the damage site, necessitating lesion pre- processing by the proteasome.

Our data suggest that the proteasome is not involved in DPC removal by HR. It is conceivable that pre-processing by a protease such as SPRTN (59) is required. However, Desphande *et al.* demonstrated that addition of purified recombinant MRN complex to a DNA-crosslinked protein substrate (generated by binding streptavidin to a biotin-labelled oligonucleotide) led to protein removal via sequential endonuclease and exonuclease processing (57). Our model therefore proposes that DPCs that are tagged with a K63-polyubiquitin chain–but which lack the K48 chain–become substrates for recombinational repair. It remains to be determined whether the DNA-crosslinked protein is subject to proteolytic processing and/or de-ubiquitination prior HR. This latter prediction, i.e. that de-ubiquitination is an obligate step in the initiation of HR repair of DPCs is consistent with literature reports that K63-linked polyubiquitin chains are key to the recruitment of HR machinery to DSBs (60–62). Our studies could not determine whether the K63 chains on the crosslinked protein are lost as a result of de-ubiquitination or as a byproduct of DPC repair by HR. However, we predict that elements of the HR machinery are responsible for deubiquitination of the DPC. This prediction is supported by literature reports that homologous recombination repair of chromosomal DNA is mediated by K63-deubiquitination by BRCC36. Interestingly, studies have shown that BRCC36 inactivation is associated with unrestrained DNA end resection and increased HR repair, suggesting that K63-deubiqitination by HR machinery limits unproductive DNA repair (63,64).

## Supplementary Material

gkad860_Supplemental_File

## Data Availability

The data underlying this article are available in the article and in its online supplementary material.
