# Supplementary material for: Ubiquitin signaling and the proteasome drive human DNA–protein crosslink repair
Source: Nucleic Acids Res. 2023 Oct 16;51(22):12174–84. doi: 10.1093/nar/gkad860 (PMC10711432; doi:10.1093/nar/gkad860)
Supplement: gkad860_Supplemental_File [file gkad860_supplemental_file.docx]

**Supplementary Figures**

**Supplementary Figure 1. DPC structure.** Site-specific DPC substrates are generated by trapping human oxoguanine glycosylase (OGG1) to an abasic site in a double stranded M13 molecule. Lysine residue 249 of OGG1 nucleophilically attacks C1, the attachment site of the deoxyribose sugar in the DNA backbone to a site specific 8-oxoguanine (8-oxo-dG) residue. Following this nucleophilic attack, 8-oxo-dG is expelled, leaving an apurinic site in the DNA. Including sodium cyanoborohydride in the reaction causes the reduction of the Schiff base intermediate formed by OGG1 during the removal of 8-oxo-dG, covalently trapping OGG1 to C1 of the deoxyribose and creating a DPC within an uncleaved DNA strand.

**Supplementary Figure 2. Schematic of SSPEqPCR assay.** Prior to qPCR analysis, DPC samples are treated to 8 cycles of damaged strand specific primer extension (SSPE). A n M13 molecule containing a polymerase blocking lesion (left) that undergoes the 8 cycles of damaged strand specific primer extension would not create any new amplicons, while an undamaged M13 molecule (right) would generate 8 new strands. This difference in the number of new strands generated after SSPE is amplified in the following qPCR analysis, in which each strand is amplified exponentially for 30 cycles. This asymmetric PCR analysis allows for the sensitive quantification of the percentage of damaged DPC molecules in DPC samples that have or have not been transfected into mammalian cells.


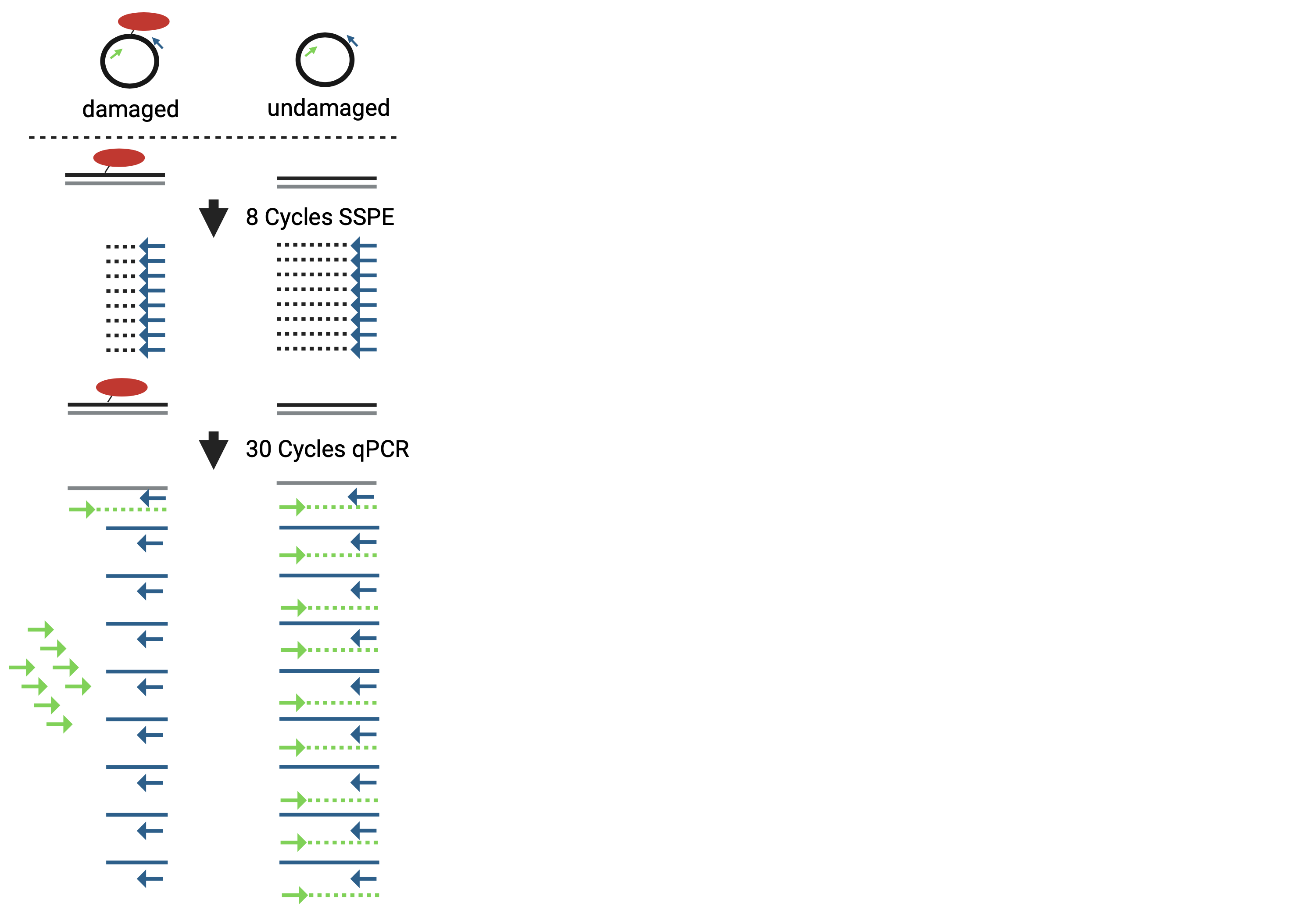


**Supplementary Figure 3. DPC removal in XPA KO cells, but not XPA wt cells, is homologous donor dependent.** A. Western blot analysis of 20ug of cell lysate collected from HT1080 cells WT or KO for XPA. B. DPCs were transfected into HT1080 cells that were wildtype for XPA or had an XPA KO, in the presence of heterologous and homologous donor. DPCs were recovered 1 hour following transfection and removal was quantified using the KCl/SDS-qPCR assay. * P=0.02

**Supplementary Figure 4. DPC removal is not affected by SPRTN deficiency.**   DPCs were transfected into SPRTN proficient (SPRTN+/+) or deficient cells (SPRTN+/-), recovered 1 hour following transfection and removal was quantified using the KCl/SDS-qPCR assay. The difference between the (+/+) and (+/-) lines is not significant, P=0.89.
